# Combined pelvic and acetabular injuries: clinical features and treatment strategies of a unique injury pattern

**DOI:** 10.1186/s13018-023-03897-0

**Published:** 2023-06-08

**Authors:** Renjie Li, Peishuai Zhao, Jianzhong Guan, Xiaopan Wang, Leyu Liu, Min Wu

**Affiliations:** Department of Orthopaedics, The First Affiliated Hospital of Bengbu Medical College, Bengbu, Anhui China

**Keywords:** Pelvic fracture, Acetabular fracture, Combined injury, Reduction

## Abstract

**Background:**

To explore the clinical characteristics of patients with unstable pelvic fractures combined with acetabular fractures and to discuss the treatment strategies for such patients to help guide treatment.

**Methods:**

We retrospectively assessed 24 patients admitted to our hospital from June 2018 to June 2022 with unstable pelvic fractures combined with acetabular fractures, including 15 male patients and 9 female patients with a mean age of 44.8 years. According to the Tile pelvic fracture classification, 15 cases were type B, and 9 cases were type C. The acetabular fractures were classified using the Letournel–Judet classification. There were 8 transverse fractures, 4 transverse and posterior wall fractures, 3 anterior and posterior hemitransverse fractures, 6 both-column fractures, 2 T-shaped fractures and 1 anterior column fracture. We recorded the cause of the patient's injury and vital signs on admission and assessed the patient's treatment strategy and prognosis.

**Results:**

All patients completed the surgery successfully, and the follow-up ranged from 6 to 42 months, with a mean of 23 months. The healing time for pelvic fractures ranged from 11 to 21 weeks, with a mean of 14.8 weeks, and the postoperative displacement of the posterior pelvic ring ranged from 1.2 to 9.0 mm, with a mean of 3.5 mm. The final clinical outcome at follow-up was evaluated using the Majeed scale: excellent in 11 cases, good in 10 cases and fair in 3 cases; the excellent rate was 87.5%. The time to healing of the acetabular fracture ranged from 13 to 25 weeks, with a mean of 15.9 weeks, and the postoperative displacement of the acetabular fracture ranged from 0.6 to 5.2 mm, with a mean of 1.9 mm. Hip function was assessed at the final follow-up using a modified Merle D’ Aubigné and Postel scale: there were 9 excellent, 11 good and 4 acceptable scores; an excellent rate of 83.3% was achieved.

**Conclusion:**

Patients with unstable pelvic fractures combined with acetabular fractures suffer severe trauma and complex mechanisms of injury. Treatment needs to be individualized, taking into account the patient's physiological status, fracture classification and degree of displacement.

## Introduction

Unstable pelvic fractures combined with acetabular fractures are a complex and challenging injury pattern that is not uncommon with the rapid growth of the transportation and construction industries. According to previous studies in the literature, the incidence of combined pelvic fracture and acetabular fracture accounts for 5.1–16.1% of all pelvic and acetabular injuries [1–5]. The difference stems mainly from the inconsistent definition of combined pelvic and acetabular injuries. Typically, when an injury occurs to the anterior pelvic ring, a fracture of the pubic branch involving the low anterior wall of the acetabulum is often considered to be a simple pelvic ring injury; additionally, some acetabular fractures with posterior extension of the fracture line to the sacroiliac joint are treated as simple acetabular fractures only. In summary, only pelvic ring injuries that require surgical intervention independent of that needed to achieve acetabular fracture fixation can be considered combined injuries [5]. Stable pelvic ring injuries tend to be less intrinsically linked to acetabular fractures and have less impact on the treatment of acetabular fractures; therefore, the focus of this study is on patients with unstable pelvic fractures combined with acetabular fractures.

The treatment of unstable pelvic fractures focuses on restoring stability to the pelvic ring while maintaining stable vital signs, allowing early movement and reducing the risk of complications, while acetabular fractures, as intra-articular fractures, achieve anatomical repositioning of the articular surface as the ultimate goal of surgery [6]. The two treatment strategies are different but anatomically inextricably linked, and the fixation of one injury is likely to have a direct impact on the fixation of the other. Patients with combined pelvic and acetabular injuries that are not well treated are prone to a poor prognosis of chronic sacrococcygeal pain, bilateral lower limb inequality, traumatic arthritis of the hip and femoral head necrosis, which can seriously affect the quality of life of patients [7, 8]. There are few studies on combined pelvic and acetabular injuries, and there is no clear consensus on the principles of treatment.

The main objective of this study is to assess the clinical characteristics of patients with unstable pelvic fractures combined with acetabular fractures and to discuss treatment strategies for such patients to help guide treatment.

## Patients and methods

We retrospectively analyzed 335 patients with pelvic fractures and acetabular fractures admitted to the Department of Traumatology and Orthopaedics of the First Affiliated Hospital of Bengbu Medical College from June 2018 to June 2022, of whom patients with unstable pelvic fractures combined with acetabular fractures were selected. The following were the inclusion criteria: 1. age > 14 years; 2. stable posterior pelvic ring injury due to trauma (Tile B or C injury [9]); and 3. surgically treated acetabular fracture. Exclusion criteria included the following: 1. age < 14 years; 2. stable pelvic ring fracture (Tile A injury [9]); and 3. relevant contraindications to surgery or anaesthesia and not undergoing surgical treatment. Thus, we identified 24 (7.2%) patients who met the inclusion criteria for unstable pelvic fractures combined with acetabular fractures.

We recorded and assessed patients’ sex, age, cause of injury, systolic blood pressure on admission, injury severity score (ISS), Glasgow Coma Score (GCS), blood transfusion within 24 h of injury, fracture typing, time from admission to surgery, sequence of reduction, quality of reduction, postoperative complications and prognosis. These indicators allowed us to compare our data with previously reported outcomes. All patients were given supracondylar femoral traction on admission, and multi-angle X-rays of the pelvis (pelvic front view, inlet view, outlet views, iliac oblique view, and obturator oblique view) and thin-section CT (0.625 mm) were collected, whereby the type of fracture of the patient was determined. Pelvic fractures are classified according to the Tile classification [9] and the Young–Burgess classification [10], and acetabular fractures are classified according to the Letournel–Judet classification [11]. The classification of fractures was determined by a joint consultation between two experienced orthopaedic trauma surgeons.

### Surgical treatment

**Fig. 1:**
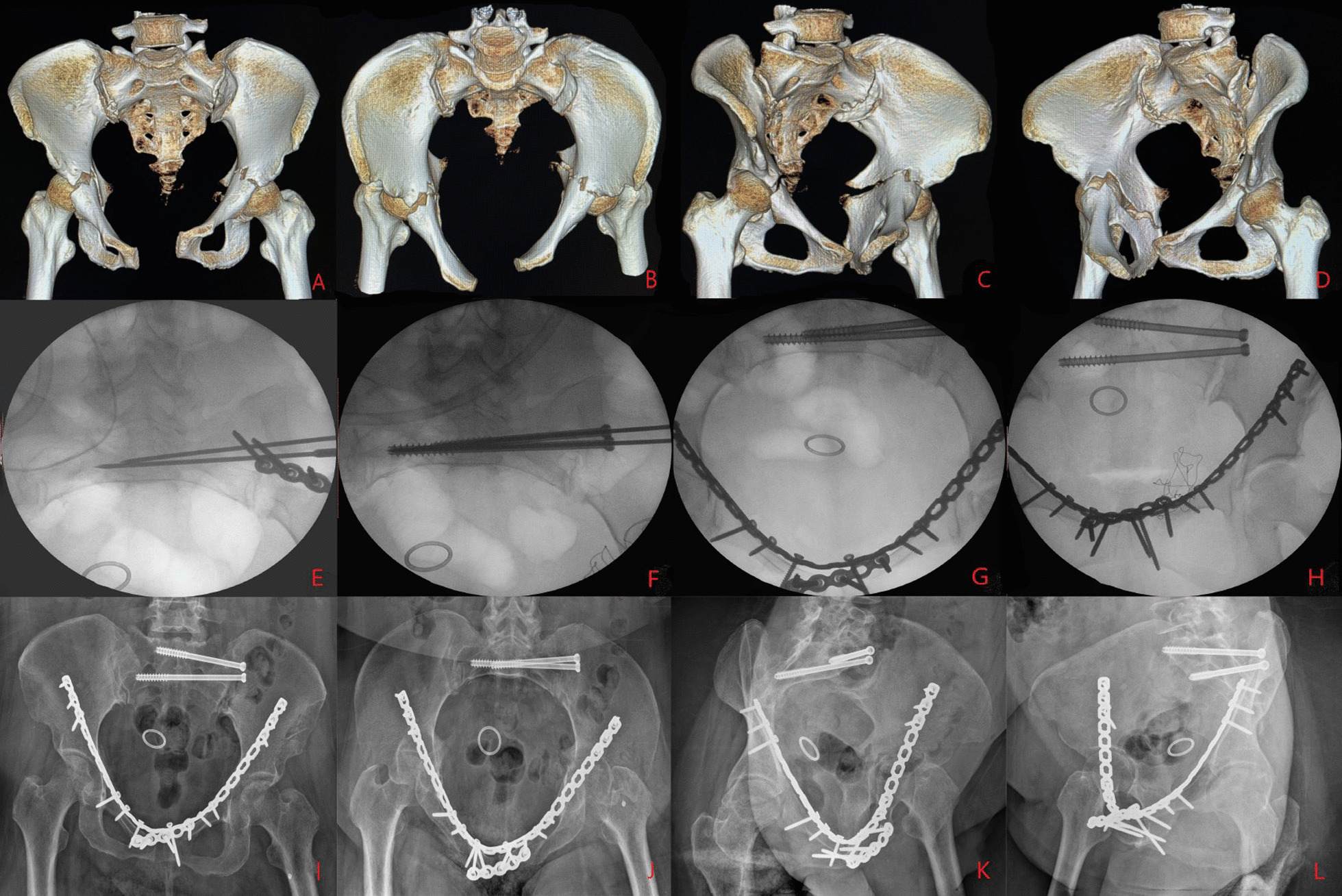
A 56-year-old female patient had an APC III pelvic fracture combined with bilateral transverse acetabular fractures due to a motor vehicle accident (**A**, **B**, **C**, **D**) and underwent surgery on the 6th day after the injury. A modified Stoppa approach with a small incision in the left iliac fossa was used to first reposition the sacroiliac joint and temporarily fix it with a small plate (**E**), followed by the placement of two sacroiliac screws (**F**) and finally repositioning of the bilateral acetabulum with an additional small incision in the right iliac fossa (**G**, **H**). The postoperative X-ray film showed excellent reduction in the pelvic fracture and good reduction in the acetabular fracture. The X-ray at half a year (**I**, **J**, **K**, **L**) after surgery showed fracture healing

**Fig. 2:**
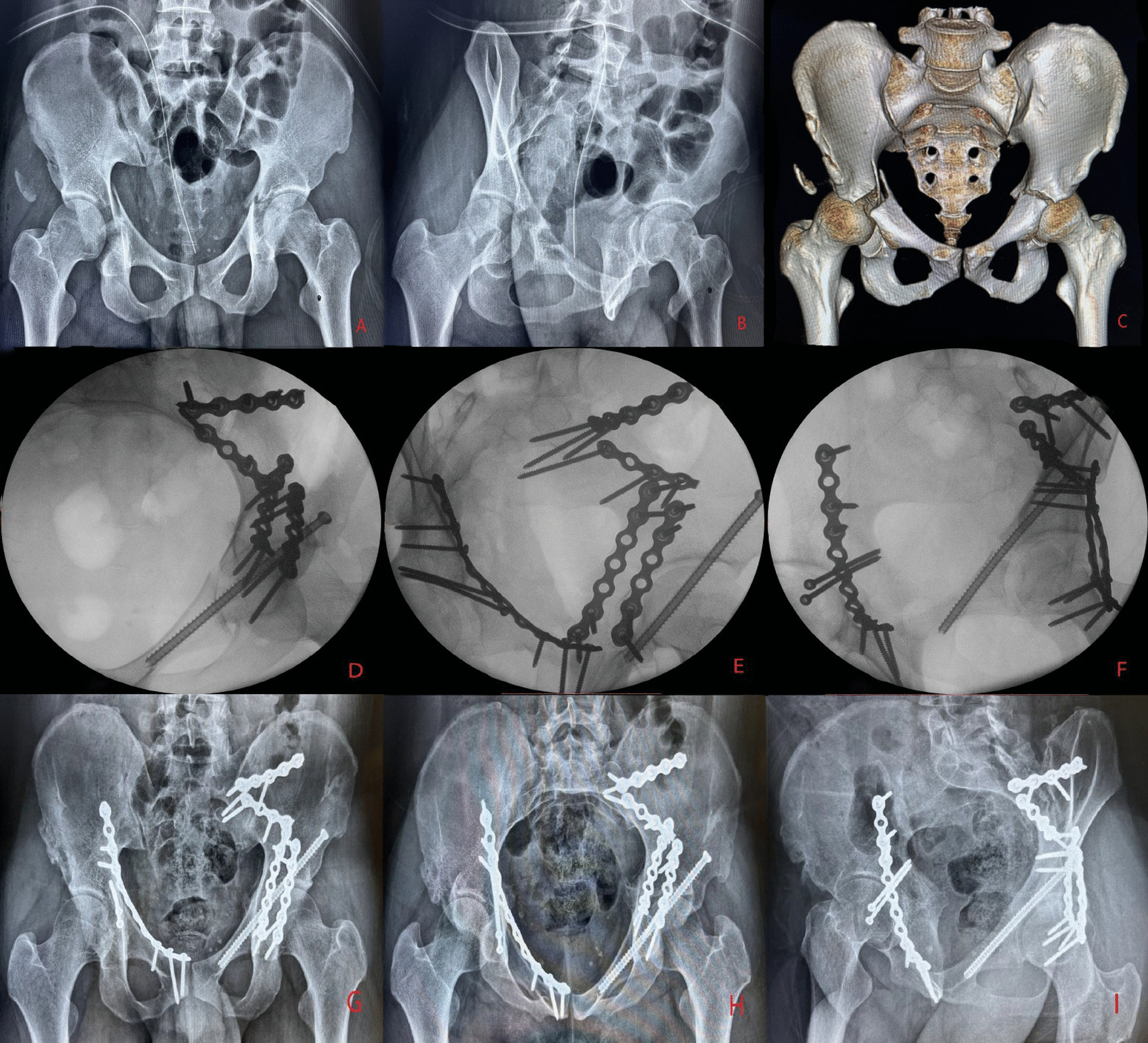
A 25-year-old male patient suffered a Tile C pelvic fracture with bilateral transverse acetabular fractures as a result of a motor vehicle accident and received his first surgical treatment on the 10th day after the injury (**A**, **B**, **C**). The sacroiliac joint was first repositioned in the supine position with a left pararectus approach and fixed with double plates (**D**). Then, the left acetabulum was repositioned and fixed with the K-L approach (**E**), and the anterior column of the acetabulum was fixed with an antegrade anterior column screw. Considering that the patient had more intraoperative bleeding, the right pararectus approach was performed to fix the contralateral acetabulum 4 days later (**F**). The X-ray 1 year after surgery showed anatomical reduction in the pelvis and acetabulum with good fracture healing and no screw breakage or entry into the acetabulum (**G**, **H**, **I**)

**Fig. 3:**
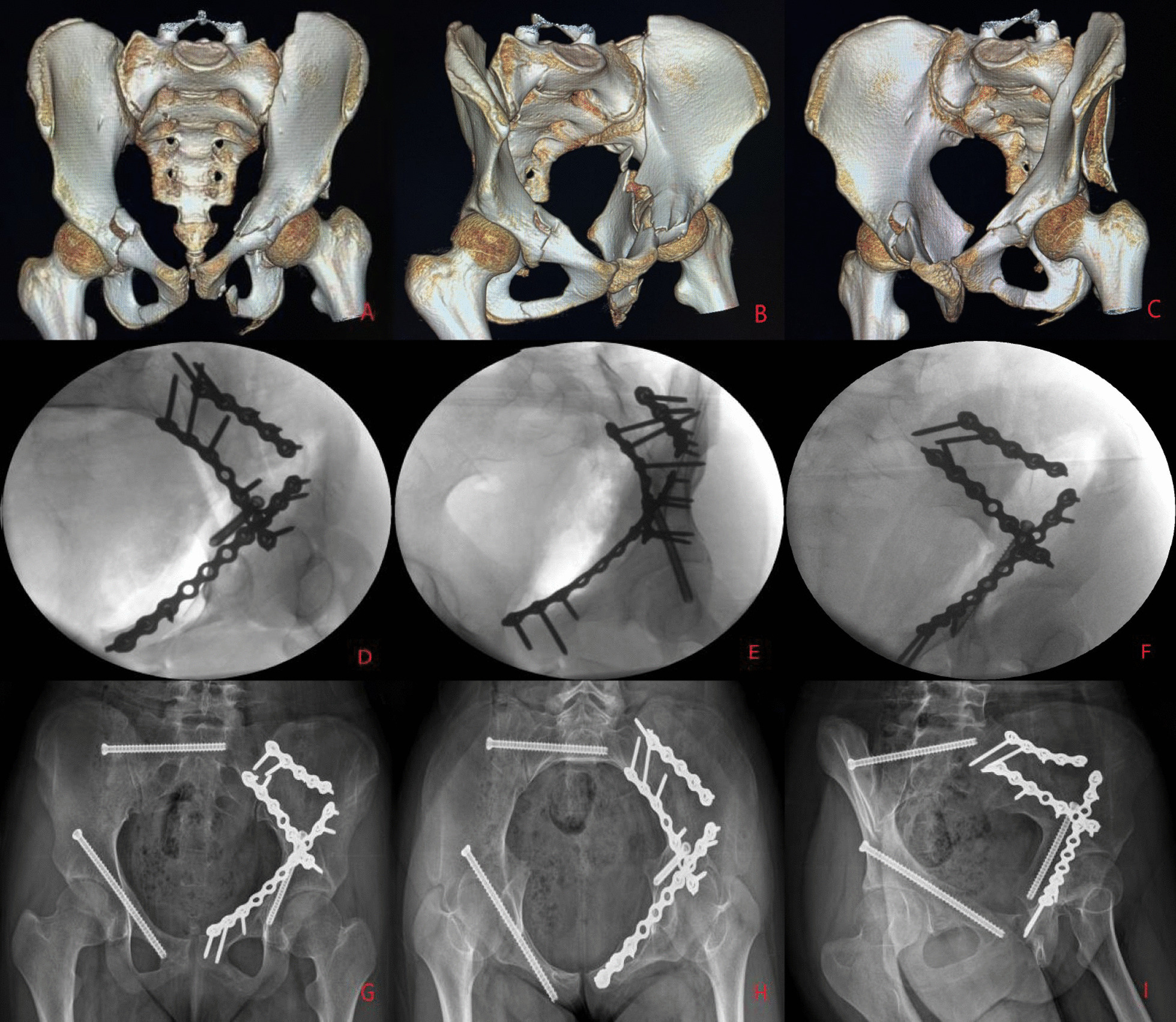
A female patient, 19 years old, sustained a Tile B pelvic fracture with a left both-column fracture as a result of a motor vehicle accident and was treated surgically on the 6th day after the injury (**A**, **B**, **C**). The left acetabulum was repositioned and fixed by a pararectus approach first (**D**), then the right closed repositioned sacroiliac screw was placed, and finally, the right antegrade anterior column screw was placed (**E**, **F**). One year after surgery, the fracture healed well, and hip function was excellent (**G**, **H**, **I**). No complications, such as loosening of the internal fixation, heterotopic ossification formation or femoral head necrosis, were observed

All operations were performed under general anaesthesia after the patient's vital signs had stabilized, and the choice of surgical approach and sequence of reduction was individualized. For patients with a significantly displaced acetabular fracture that could not be fixed percutaneously and who had a significantly displaced ipsilateral sacroiliac joint, we chose the pararectus approach [12]. The posterior pelvic ring is reduced under direct vision from window 2 and reinforced with double plates or double sacroiliac screws, followed by traction reduction and strong fixation of the acetabular fracture. For patients with a less displaced acetabular fracture that is feasible for closed reduction, we consider closed reduction percutaneous hollow screw fixation of the acetabular fracture before treating the pelvic ring injury. For patients with posterior pelvic ring injuries where closed reduction percutaneous hollow screw fixation is feasible, we prefer a single pararectus approach [12] for anatomical reduction in acetabular fractures first, combined with a small iliac fossa incision if necessary. In the case of combined posterior acetabular wall or posterior column fractures, a combined anterior–posterior approach can be adopted, with an anterior approach via the paramedian approach [12] or a modified Stoppa approach [13] and a posterior Kocher–Langenbeck approach [14]. In cases where the mechanism of injury is unclear and there is no intrinsic link between the pelvic and acetabular injuries, such as when the posterior pelvic ring is not on the same side as the acetabular fracture, treatment can be carried out separately depending on the severity of the injury. Fixation of anterior pelvic ring injuries is often considered last (Figs. 1, 2 and 3).

### Postoperative management

Postoperative deep wound drains were placed for 2–4 days, and routine anticoagulants were given to prevent thrombosis. Multiangle radiographs (pelvic front view, inlet view, outlet view, iliac oblique view, and obturator oblique view) and thin-section CT (0.625 mm) were performed within one week after surgery. Regular follow-up visits were made to assess the patient's recovery and to guide functional exercises. At 8 weeks postoperatively, patients were allowed to walk using crutches, and at 12 weeks, depending on the healing of the fracture, they were allowed to attempt to walk without crutches.

### Statistical analysis

Routine photographic evaluation was performed at 1, 3, 6, 12 and 24 months postoperatively. Pelvic fractures were evaluated according to the Matta and Tornetta scale [15]. The quality of the reduction was evaluated according to the maximum distance of fracture displacement on pelvic radiographs in three positions (pelvic front view, inlet view, and outlet views) within one week after surgery, with ≤ 4 mm being considered excellent, 5–10 mm good, 10–20 mm moderate and more than 20 mm poor. The Majeed functional scale [16] was used to assess functional outcome according to pain, sitting and standing, ability to work, sexual ability, assisted walking, gait and walking distance, with a score of ≥ 85 being considered excellent, 70–84 good, 55–69 acceptable and < 55 poor. The quality of postoperative repositioning of acetabular fractures was assessed using the Matta criteria [17], which classified the quality of reduction as anatomical (0–1 mm displacement), satisfactory (2–3 mm displacement) and poor (> 3 mm displacement) according to the degree of fracture displacement on radiographs and CT within one week after surgery. The clinical outcome of the treatment of acetabular fractures was assessed at the final follow-up according to the modified Merle D’ Aubigné and Postel scale [18], which evaluates hip function according to pain, walking and range of motion, with 18 points for excellent, 15–17 points for good, 13–14 points for acceptable and < 13 points for poor.

## Results

### Clinical features of the patient

There were 15 male patients and 9 female patients in this study, with a mean age of 44.8 years (17 to 72 years). The most common cause of injury was a car accident (15 cases), followed by a fall from a height (6 cases) and a smash injury from heavy objects (3 cases). The mean systolic blood pressure on admission was 114.3 mmHg (range 75–152 mmHg), the mean ISS score was 17.75 (range 10–28), the mean GCS score was 13.9 (range 9–15), and the mean red blood cell input within 24 h postinjury was 1.6 u (range 0–8 u), with four (16.7%) patients receiving more than 5 u of red blood cells within 24 h postinjury.

### Fracture classification

Of the 24 patients with pelvic ring injuries, 15 were type B and 9 were type C according to the Tile classification [9], 11 were lateral violence type (LC), 10 were anterior–posterior compression type (APC) and 3 were vertical shear type (VS) according to the Young–Burgess system [10]. The acetabular fracture typing was based on the Letournel–Judet typing [11], with transverse acetabular fractures (8 cases) being the most common, followed by both-column fractures (6 cases), transverse and posterior wall fractures (4 cases), anterior and posterior hemitransverse fractures (3 cases), T-shaped fractures (2 cases) and anterior column fractures (1 case).

### Treatment results

All patients completed the operation successfully, and there were no deaths. The mean time from injury to surgery was 10.3 days (range 4 to 24 days), and the mean length of stay was 9.6 days (range 7 to 37 days). Postoperative complications included deep vein thrombosis of the lower limbs in three cases; numbness due to sciatic nerve injury in one case; and fat liquefaction of the incision in three obese patients, two of whom healed with dressing changes and one of whom had incision infection, which healed after resurgical debridement and irrigation. Follow-up time ranged from 6 to 42 months, with a mean of 23 months.

The mean healing time of the pelvic fractures was 14.8 weeks (range 11–21 weeks), and the mean displacement of the posterior pelvic ring was 3.5 mm (range 1.2–9.0 mm), with 11 cases of excellent, 10 cases of good, 2 cases of moderate and 1 case of poor reduction according to the Matta and Tornetta scale [15]. The final clinical outcome at follow-up was evaluated using the Majeed scale [16], with 11 excellent, 10 good and 3 fair cases; the excellent rate was 87.5%. The mean time to healing of the acetabular fracture was 15.9 weeks (range 13–25 weeks), and the mean postoperative displacement of the acetabular fracture was 1.9 mm (range 0.6–5.2 mm). According to the Matta scale [17], anatomical reduction in the acetabulum was achieved in 8 patients, satisfactory repositioning in 12 cases and unsatisfactory reduction in 4 patients. Hip function was assessed at the final follow-up using the modified Merle D’ Aubigné and Postel scoring criteria [18], with 9 excellent scores, 11 good scores and 4 acceptable scores; an excellent rate of 83.3% was achieved.

## Discussion

According to previous studies in the literature, the incidence of pelvic fractures combined with acetabular fractures accounts for 5.1%-16.1% of all pelvic and acetabular injuries[1–5]. In the present study, we excluded stable pelvic ring injuries, resulting in a 7.2% incidence of unstable pelvic fractures combined with acetabular fractures. Most of these patients had a high-energy injury, which usually represents a higher severity of injury and mortality, whereas in the study by Tibbs et al. [19], mortality was lower in patients with pelvic fractures combined with acetabular fractures (10.6%) than in patients with simple pelvic fractures (16.5%). There were no fatal patients in this study, and although the patients’ ISS scores were close to those of patients with simple pelvic fractures in Tibbs’ study, the admission blood pressure and the number of red blood cells received within 24 h of admission were less severe in response to the severity of the condition than in pelvic fracture patients, which to some extent reflects the magnitude of mortality. The reduction in mortality in patients with combined injuries compared to those with simple pelvic fractures may be related to its mechanism of injury, where violence to the pelvic ring may provide some protection to soft tissues such as peripheral vascular nerves through the energy dissipation of the acetabular fracture.

According to previous studies in the literature, fractures of the posterior acetabular wall caused by direct violence are more common [20]. In contrast, Suzuki et al. [2] suggested that the incidence of transverse acetabular fractures was as high as 61.2% in patients with combined pelvic and acetabular injuries. The most common type of injury was a transverse acetabular fracture associated with an injury to the anterior aspect of the ipsilateral sacroiliac joint. In the present study, transverse fractures were also the most common type of fracture, followed by both-column fractures. The mechanism of injury in patients with combined injuries is complex. Letournel [11] suggested that direct lateral trauma is the most common cause of injury in combined pelvic and acetabular injuries, but recent studies have found a similar incidence of pelvic APC-type injuries and LC-type injuries [5], which was confirmed in our study. One possible explanation for the mechanism of injury in combined pelvic and acetabular injuries is that the high-energy injury leading to the transverse acetabular fracture acts on the ilium, and as the acetabulum is connected to the upper part of the ilium, the force may further disrupt the sacroiliac joint through the force of external or internal rotation of the acetabular surface, ultimately leading to injury to the posterior ring of the ipsilateral pelvis.

The treatment of patients with combined pelvic and acetabular injuries requires planning based on the patient's physiological status and other concomitant injuries. Proper preoperative planning is critical to the patient's prognosis, including the choice of operative time, intraoperative position, surgical approach and reduction sequence [5]. Due to the deep anatomical position of the pelvis and acetabulum and the surrounding muscles and ligaments being relatively hypertrophic, we found in this study that even with effective preoperative lower limb traction, the difficulty of surgical reduction and fixation increases with delayed surgery due to scab formation and soft tissue adhesion contractures. Vallier et al. [21] retrospectively assessed the clinical data of 645 patients with acetabular or pelvic fractures, including 40 patients with combined pelvic and acetabular injuries, and they observed a lower incidence of acute respiratory distress syndrome, fewer pulmonary complications and a lower incidence of multiorgan failure in patients who underwent surgery within 24 h. However, in clinical practice, as most injuries originate from high-energy violence, patients are often accompanied by trauma to other sites, such as the chest and abdomen, and are in poor general physical condition, making it difficult to perform surgery in a timely manner. In general, 4–7 days after injury is a good time to operate or as early as possible if closed internal fixation of the posterior pelvic ring is feasible [5].

Combined pelvic and acetabular injuries are difficult to perform, requiring the surgeon to be skilled in the various surgical approaches and reduction methods for pelvic and acetabular fractures. The intraoperative position must take into account not only the fracture type but also the physiology of the patient and other injuries, for example, avoiding the prone position when there are multiple rib fractures or lung injuries. A single surgical approach should be taken whenever possible, such as a pararectus approach [12] or an iliac inguinal approach [22], which allows simultaneous access to the posterior pelvic ring and acetabulum and allows for timely adjustment of the reduction, which helps to improve the overall outcome [5].

There is no clear consensus on the order of reduction for combined pelvic and acetabular injuries. Suzuki et al. [2] suggested that the degree of displacement of the posterior pelvic ring is an important predictor of acetabular fracture displacement, and good initial reduction in the posterior pelvic ring will facilitate subsequent acetabular reduction; therefore, they suggested that reduction and fixation of the posterior pelvic ring should be prioritized over acetabular fractures, while Halvorson et al. [5] suggested that in some cases, it may be difficult to obtain an anatomical reduction in the posterior ring, either with minimally invasive screws or open surgery, due to other injury factors, and priority should be given to resetting and fixing the acetabulum at this time. Vaidya et al. [7] concluded that for patients with transverse, T-shaped and posterior wall fractures of the acetabulum that can be treated with a single K-L approach [14], reduction and fixation of the pelvic ring may assist in the reduction in the acetabular fracture, and placement of an INFIX device in the anterior ring after posterior ring fixation will help to maintain stability of the pelvic ring without compromising subsequent repositioning of the posterior acetabular column. In this study, we took an individual approach to treatment, carefully assessing patients on preoperative X-ray and CT, and we referred to the following principles for the selection of the order of reduction: 1. In general, a displacement of the posterior pelvic ring of less than 1 cm is acceptable [23, 24], whereas the displacement of the weight-bearing area at the top of the acetabulum should be less than 2 mm [25–27]. Therefore, we sought more anatomical reduction in the acetabulum during treatment. 2. According to previous studies in the literature, the posterior pelvic ring provides the main stability of the pelvis (60–70%), and the anterior pelvic ring provides 30–40% of the stability of the pelvis [28]; therefore, we tend to treat injuries to the anterior pelvic ring last. 3. For complex acetabular fractures, such as both-column fractures, when combined with significant displacement of the ipsilateral posterior pelvic ring, we prefer to reset and fix the posterior pelvic ring under direct vision via a pararectus approach [12] or an iliac-inguinal approach [22]. This facilitates the search for a suitable anatomical reference mark during acetabular fracture reduction, and the precise reduction in the posterior pelvic ring will facilitate subsequent acetabular reduction [2]. The stability of the posterior pelvic ring needs to be enhanced intraoperatively by fixing the posterior pelvic ring with double sacroiliac screws or double plates to avoid redisplacement of the pelvic ring after traction and reduction in the acetabular fracture.

## Conclusions

In conclusion, unstable pelvic fractures combined with acetabular fractures are a complex type of injury, and there are no uniform diagnostic and classification criteria, nor is there a uniform surgical procedure. Due to the complexity of the mechanism of injury, a case-by-case analysis is needed, taking into account the patient's physiological status, fracture classification and degree of displacement to achieve the best prognosis for these patients.

## Data Availability

The datasets used and/or analyzed during the current study are available from the corresponding author on reasonable request.

## Abbreviations

ISS: Injury severity score
GCS: Glasgow coma score
CT: Computed tomography
K–L: Kocher–Langenbeck
LC: Lateral compression
APC: Anterior–posterior compression
VS: Vertical shear
